# Factors that influence dietary behavior toward iron and vitamin D consumption based on the theory of planned behavior in Iranian adolescent girls

**DOI:** 10.1186/s12937-019-0433-7

**Published:** 2019-02-06

**Authors:** Ali Alami, Seyedeh Belin Tavakoly Sany, Elaheh Lael-Monfared, Gordon A Ferns, Maryam Tatari, Zahra Hosseini, Alireza Jafari

**Affiliations:** 1 Department of Public Health, School of Public Health, Social Determinants of Health Research Center, Gonabad University of Medical Sciences, Gonabad, Iran; 2 Social Determinants of Health Research Center, Mashhad University of Medical Sciences, Mashhad, Iran; 3 Department of Health Education and Health Promotion, Faculty of Health, Mashhad University of Medical Sciences, Mashhad, Iran; 4 Student Research Committee, Department of Public Health, School of Health, Shahid Beheshti University of Medical Sciences, Tehran, Iran; 5 Department of Medical Education, Brighton and Sussex Medical School, Division of Medical Education, University of Brighton Falmer campus, Brighton, BN1 9PH UK; 6 School of Health, Torbat Heydariyeh University of Medical Sciences, Torbat Heydariyeh, Iran; 7 Department of health, Social Determinants of Health Research Center, Gonabad University of Medical Sciences, Gonabad, Iran; 8 Social Determinants of Health Research Center, Gonabad University of Medical Sciences, Gonabad, Iran; 9 Social Determinants of Health Research Center, Mashhad University of Medical Sciences, Mashhad, Iran

**Keywords:** Iron and vitamin D supplement, Theory of planned behavior, Female student, Adolescents, Structural equation modeling

## Abstract

**Background:**

The impact of iron and vitamin D supplements among adolescent is not well understood. The prevalence of supplement use, and the behavioral intentions of adolescents was studied to better understand the data on supplement intake. We used the theory of Planned Behavior (TPB) to explore the determinants that influence supplement intake, and its potential constructs to examine determinants that influence dietary supplement behavior towards the use of iron and vitamin D supplements amongst a sample of Iranian schoolgirl.

**Methods:**

This was a cross-sectional study of 485 adolescent girls aged 12–17 years. Multiple analytical models including hierarchical regression and structural equation modeling (SEM) were used to examine the association between TPB constructs and adolescent’s behavioral intentions to consume dietary supplements.

**Results:**

Based on the results of SEM, constructs of TPB and knowledge were found to predict 74% of the variation in the behavioral intentions of the schoolgirls. SEM indicated that perceived behavioral control (PBC) and knowledge had significant associations with intention behaviors to take nutritional supplements.

**Conclusion:**

TPB and its constructs were used to establish the determinants of iron and vitamin D intake among schoolgirls in Iran. This outcome indicates that efforts to promote behavioral intentions through targeting subjective norms, attitude, and PBC may promote supplement use.

## Background

Children who are entering adolescence need to consume adequate dietary micronutrient because they are going through many significant physical and intellectual changes [1, 2]. Several studies have reported that adolescents who consume a diet rich in micronutrients, have a lower risk of and cardiovascular diseases than those with a dietary pattern that is high in animal proteins, carbohydrates and fats, and lower in micronutrients [3–5]. The transition to adulthood is also considered to be a critical time for dietary intervention to improve long-term health behavior and responsibility for diet [6]. Previous studies have indicated that dietary interventions can improve long-term health behavior and lead to periodic changes in nutritional intakes and dietary behaviors in adulthood [7–9].

Deficiencies of iron and vitamin D are common in the adolescent population, which can have a negative impact during phases of rapid growth, increasing susceptibility to infection, autoimmune and other chronic disease and also impair mental development and learning [10, 11]. Dietary iron is mostly present in animal products (e.g red meat, fish, egg), certain plant foods and legumes. The main sources of vitamin D include dairy products, egg yolks, seafood and fruit juices. Numerous studies have identified an association between poor iron status and low Vitamin D concentration. Vitamin D deficiency is associated with an increased risk of deterioration of iron status and anemia [12–14]. It has been hypothesized that vitamin D may influence iron metabolism and erythropoiesis, while iron, in turn, is essential for the synthesis of vitamin D [12, 13]_._

According to the National Family Health Survey in Iran, 14–18% of girls suffer from iron deficiency anemia and 31% were reported to have insufficient iron stores [15]. In Iran, 14% of adolescents under the age of 18 suffer from iron deficiency anemia [16–19]. Current estimates indicate that vitamin D insufficiency/ deficiency is a common health problem, affecting 1 billion people globally, and 40–100% of European and American elderly men and women suffer from these deficiencies [20, 21]. The results of the meta-analysis study in 2018 in Iran indicated that the prevalence of vitamin D deficiency in men, pregnant women, and all women was respectively 46, 60, and 62% [22]. Whilst there may be sufficient levels of sunlight in Iran, the high prevalence of vitamin D deficiency may due to the types of clothing worn, dietary habits (low consumption of dairy products and seafood), lifestyle, air pollution, and skin pigmentation [21]. Numerous studies have shown that prevalence of vitamin D deficiency among women population is higher than for men in Iran [23–25]. This has been explained in terms of social obligations and cultural aspects. In Iran, women and girls (age > 9 years) are instructed to dress with full body covering that is usually dark and solid in coloration. This degree of covering allows for the entire face to be revealed, while continuing to cover the hair by wearing a scarf. Studies have indicated that skin covering is an important risk factor for vitamin D deficiency because it limits exposure to the sun and the level of vitamin D absorption [23–25].

To combat vitamin D and Iron deficiency in Iranian adolescents, a national program of supplements of iron and vitamin D is being conducted routinely in Iranian high schools for female students by the Health ministry. According to this program, 16 iron pills (Iron (II) fumarate), and 9 pearl vitamin D3 are given to students weekly and monthly, respectively. Despite, several studies that have shown that the positive effect of this supplementation programs (WIFS) in reducing the rate of vitamin D and Iron deficiency, a national report showed that the prevalence of anemia and vitamin D deficiency among Iranian adolescents remains high, and this may be explained by the refusal of adolescents to take these supplements [15, 22, 26–28]. Although efforts have been made to modify supplementation programs in Iran, the effect of behavioral intentions and attitude toward Iron and vitamin D intake have not been studied systematically, especially with respect to high-risk groups. It is still unclear what/how potential determinates can affect/impact adolescent’s attitude and intention to regular intake Iron and vitamin D [15, 16, 29, 30].

In recent decades, theories of public health have been developed to improve the health promotion behaviors to use food supplements among adolescent [31]. The theory of planned behavior (TPB) is a practical theoretical perspective to evaluate factors influence individual’s decision to engage in an specific behavior [32–34]. This theory links an individual’s attitude and behavior, and states that attitude and belief toward behavior, PBC, and subjective norms, together shape an individual’s intentions to engage actual behavior at specific place and time [35–38]. Therefore, in this study, we hypothesized that TPB could be a fundamental framework to identify and explain the main determinants that may affect iron and vitamin D supplement intake in Iranian girls. With a view to providing a basis for future studies, we aimed to (1) test the fit of the TPB; (2) to explore potential determinants that influence dietary behavior toward iron and vitamin D supplementation in female adolescents; and (3) to estimate the extent of the relationship between supplementation knowledge, attitude, subjective norms, and perceived control of behavior and intention towards intake of vitamin D and iron supplements.

## Methods

### Study population and sampling technique

This cross sectional study was conducted on 485 female high school students from Gonabad city (a city in the southeastern province of Khorasan Razavi, Iran) in 2017. We divided the city into 8 regions based the existence of high schools and population density. We then used random sampling to select 16 schools from 32 school and all students in these selected high schools were considered for recruitment if they (a) were able to complete all questionnaires; (b) had normal physical health; (c) could speak and read Persian language (their native language); and (d) lived in Gonabad city over the previous six months. Students were excluded if they unwilling to participate or were unable to give informed consent; had suffered mental disturbance, upper limb disability, and visual impairment. A total of 485 student age 12–17 were involved in this study. Among all participant invited to take part, 5 refused to participate and 480 students were finally included in the data analysis. We explained any unclear questions and informed all participants concerning the aim of this survey. All eligible students gave written informed consent.

### Data collection

Data was collected by questionnaires that consisted of three parts: a) Demographical properties questionnaire b) Knowledge evaluation questionnaire c) supplement assistance of iron and vitamin D using the constructs of the TPB questionnaire.

The evaluation and validation of the questionnaire was conducted based on the comments of the group of experts (3 nutrition experts, and 5 health education experts), Content Validity Ratio (CVR) and Content Validity Index (CVI) were calculated. The CVR and CVI of the questionnaire were 0.90, which was acceptable. The questionnaire was used in a pilot study of 50 students to make sure that the questions were clear and understood by the girls, and the average of Cronbach’s alpha was 0.88 for all the structures of the planned behavior theories which was acceptable.

The questionnaire of demographic factors included student’s age, parents’ age, residence, and the parents’ employment status and education level, were reported by the participants in a self-reported questionnaire.

We designed a new questionnaire to examine the effect of potential constructs of TPB and knowledge on adolescent consumption behaviors. This questionnaire consisted of 27 questions that examined knowledge and items from the TPB (Azjen, 1991). To measure the level of knowledge, 9 questions were designed to examine what adolescent knew about the importance of iron and vitamin D supplements, and their effect on health outcome. These question were answered using a two-dimensional scale (right = 1, wrong = 0) [37, 39, 40].

The last 18 questions examined attitude, PBC, subjective norms and behavioral intention together affect Iron and vitamin D intake. To measure these constructs, the Likert scale had five options from strongly agree to strongly disagree. Further details related to questionnaire and their items were summarized into Table 1.

**Table 1:** Component loading of the adolescence dietary supplement knowledge and behaviors based on theory of planned behavior

Construct	α Cronbach coefficient (All = 0.72)	Items	Mean (SD)	Mean (SD)	Range
Knowledge [40, 55–57]	0.63	Iron supplementation helps prevent anemia	0.41 (0.38)	4.58 (2.09)	0–9
		Vitamin D supplementation helps to absorb calcium in the body	0.86 (0.47)		
		Vitamin D supplementation helps prevent rickets	0.51 (0.49)		
		Vitamin D supplementation helps prevent osteoporosis	0.57 (0.49)		
		The use of iron supplementation prevents delayed physical growth	0.23 (0.42)		
		The use of iron supplements prevents the reduction of learning power	0.56 (0.43)		
		Iron supplementation increases the Intelligence Quotient (IQ)	0.65 (0.49)		
		The use of iron supplementation prevents infections in the body	0.39 (0.48)		
		The use of iron supplementation increases the immunity of the body	0.36 (0.47)		
Attitude[50, 58]	0.66	I believe that iron and vitamin D deficiency in my body do not cause serious health problems	3.71 (1.42)	12.45 (3.43)	4–20
		In my opinion, I get enough iron and vitamin D through food, and I do not need to intake supplements	3.84 (1.21)		
		In my opinion, the use of iron and vitamin D supplements are harmful to my body and should be provided through food intake	3.18 (1.32)		
		I feel that taking iron and vitamin D helps supplements promote my health, but they are not necessary	2.51 (1.26)		
Subjective norms[50, 58]	0.65	My father disagrees that I intake iron and vitamin D supplements	3.51 (1.30)	21.70 (4.29)	6–30
		My mother agrees that I intake iron and vitamin D supplements	3.98 (1.19)		
		My friends disagree that I intake iron and vitamin D supplements	3.17 (1.22)		
		I think that my teachers disagree with the consumption of iron and vitamin D supplements	3.54 (1.29)		
		My sister agrees with the consumption of iron and vitamin D supplements	3.81 (1.05)		
		I think that my brother agrees with the consumption of iron and vitamin D supplements	3.67 (1.13)		
Perceived behavior control [50, 58]	0.60	Even if my parents, friends, and teachers disagree with the consumption of supplements, but I use iron and vitamin D supplements	3.54 (1.34)	17.43 (3.99)	5–25
		I have access to supplements at any moment	4.03 (1.19)		
		Even, supplements lead to nausea, but I still intake it	3.23 (1.29)		
		Even, supplements do not have a good taste, I still prefer to use supplements to promote my health and prevent the illness	3.09 (1.44)		
		I received iron and vitamin D supplements in free of charge from school	3.51 (1.30)		
Intention[50, 58]	0.65	I decided to start the supplements consumption next month	3.99 (1.18)	10.95 (3.05)	3–15
		This year, I would like to start supplements consumption	3.36 (1.42)		
		I’m going to start the supplements consumption this week	3.60 (1.38)		

### Statistical analyses

We used SPSS software (version 22) to analyze the data to provide descriptive data, and a multivariate model. Data were also tested by maximum likelihood estimation of a confirmatory factor analysis (CFA) method using the AMOS software for Windows, version 24. The CFA analysis allowed an assessment of the adequacy of the TPB and its constructs to present the process was surveyed and to estimate association among constructs (knowledge, attitude, perceived behavior control, subjective norms, and behavior intention). The evaluation of the model was conducted using fit criteria for the following indices: chi duo square Indicators (x^2^), Chi duo square ratio to degree of freedom (x^2^/df), root mean square error of approximation (RMSEA), adjusted goodness of fit index (AGFI), goodness of fit index (GFI), non-normed fit index (NNFI); parsimonious normed fit index (PNFI), and partnership intention (I) [41]. The model was considered to be a good fit if the (x2/df) < 5, RMSEA ≤0.08, SRMR < 0.05, AGFI > 0.8 and other indices (NFI, GFI, TLI) more than 0.9 [41, 42].

## Results

### Socio-demographic characteristics

The mean (standard deviation) for students’ age, father’s age, and mother’s age were 12.76 (0.63), 43.32 (5.64), and 39.22 (5.41) years respectively. In total, 329 (68%) of the students were residents of the city and 151 (32%) were residents of the surrounding villages. Most of parents (74%) had a diploma degree. 72% of students’ fathers were self-employed and 83% of mothers were housewives (Table 2). According to the regression model, socio-demographic variables accounted for 2.3% of the variance of behavioral intention. In this population, only residency location and father’s age were found to be associated with behavioral intention at the significance level of 0.05 (Table 3).

**Table 2:** Frequency distribution of student demographic factors

Variables		N	%
Residency location	Urban	329	68.5
	Village	151	31.5
Father’s education level	Illiterate	12	2.6
	Diploma and Under diploma	341	74.8
	Academic	103	22.8
Mother’s education level	Illiterate	15	3.3
	Diploma and Under diploma	356	77.9
	Academic	86	18.8
Father’s Job	Employee	131	27.8
	Self-employed	341	72.2
Mother’s job	Housewife	395	82.6
	Working outside the home	83	17.4

**Table 3:** The results of linear regression analysis in predicting behavioral intention of using iron supplements and vitamin D based on demographic variables

Variables	B	SE	Beta	t	*p*-value	Adjusted R Square	F	*p*-value
Age student	0.009	0.124	0.004	0.071	0.943	0.023	2.270	0.022
Residency location	0.813	0.331	0.125	2.457	0.014			
Age father	−0.107	0.035	−0.214	−3.020	0.003			
Age mother	0.086	0.039	0.162	2.224	0.027			
Father’s education level	0.497	0.481	0.069	1.034	0.302			
Mother’s education level	−0.316	0.473	−0.041	− 0.670	0.504			
Father’s Job	− 0.105	0.401	− 0.015	− 0.263	0.793			
Mother’s job	−0.053	0.426	−0.006	− 0.124	0.901			

### Model testing

Before conducting the analysis of the verified factor, the data for outliers and the normality of variables were examined, and the outlier data of each observation were analyzed by Mahalanobis distance and if necessary were omitted [43]. In the analysis of critical ratio of skewness or kurtosis of the variables, and the state of being multi- variable normal was verified, and as a result, a maximum likelihood method was conducted to estimate the parameters [44].

After performing the CFA analysis test, the knowledge (9 questions), attitude (4 questions), perceived behavioral control (PBC) (5 questions), subjective norm (6 questions) and behavioral intention (3 questions) remained in CFA. The goodness of fit for the measurement of CFA was acceptable (X2/df = 2.17, RMSEA = 0.04, AGFI = 0.89, GFI = 0.91, PNFI = 0.64, PCFI = 0.73) (Table 4).

**Table 4:** Models’ evaluation overall fit measurements

Goodness of fit indices	Confirmatory factor analysis	structural equation modelling
X^2^	312	310
df	679.66	797.66
X^2^/df	2.17	2.57
GFI	0.91	0.90
AGFI	0.89	0.87
RMSEA	0.04	0.05
PNFI	0.64	0.60
PCFI	0.73	0.67
*p*-value	*p* < 0.001	*p* < 0.001

To analyze the relationship between the four exogenous constructs (knowledge, attitude, PBC and subjective norm) and endogenous construct (behavioral intention), the structural equation modeling (SEM) was used.

**Fig. 1:**
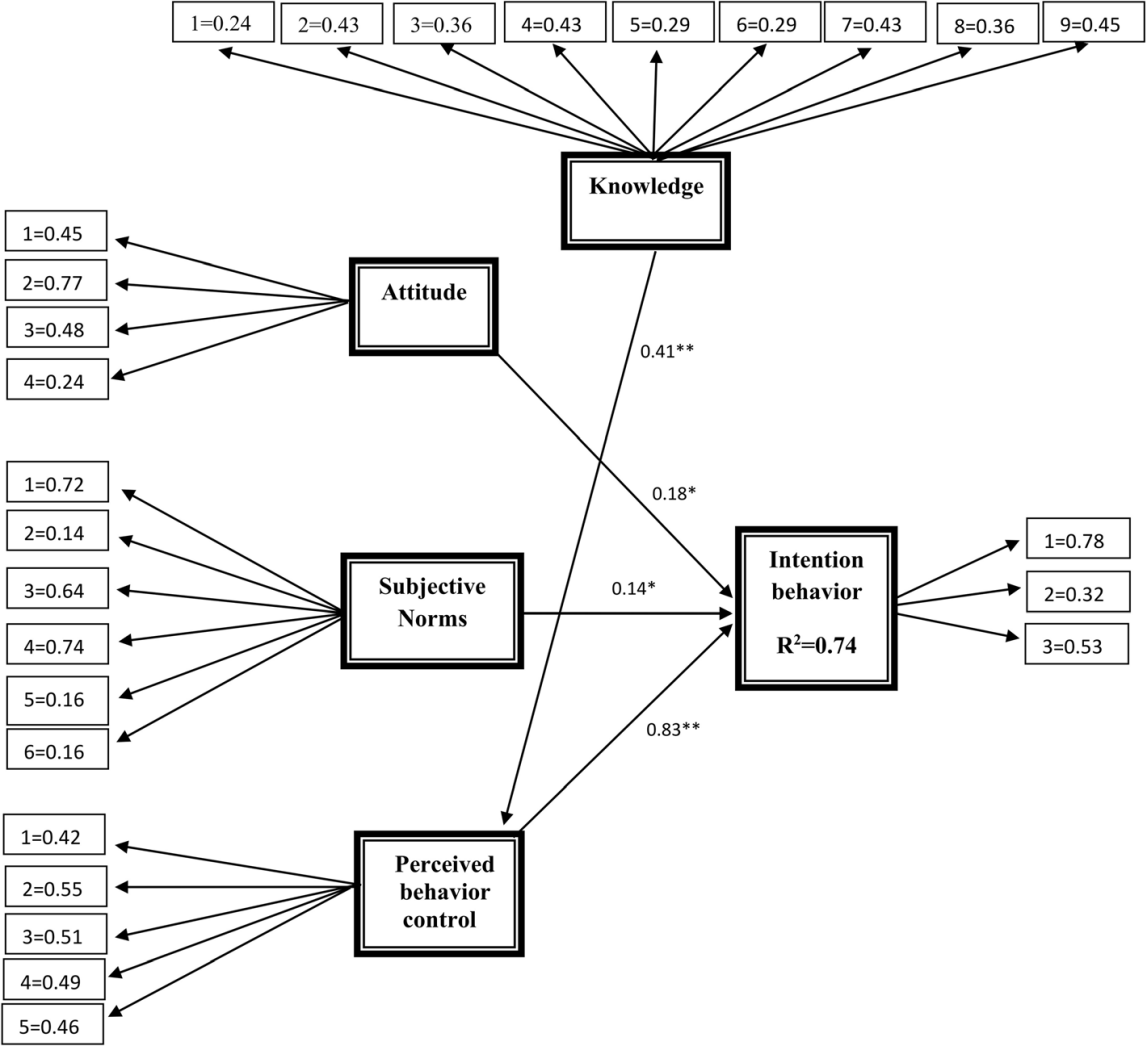
SEM and Path coefficient between variables (*p < 0.05, **p < 0.001)

The assessment of the SEM showed a good fit of indices: X2/df = 2.57, RMSEA =0.05, AGFI =0.87, GFI =0.90, PNFI =0.60, PCFI =0.67 (Table 4, Fig. 1). In summary, planned behavior theory and the knowledge structure explained 0.74 (with Confidence intervals 99% is 0.683–0.788) of the variance in the students’ behavior intention to take an iron supplement and vitamin D (Fig. 1). In summary, the results suggested that, there was significant direct effect of attitude (standardized path coefficient (SPC) =0.18, *p* < 0.05), subjective norm (SPC = 0.15, p < 0.05), and PBC (SPC = 0.83, *p* < 0.001) on behaviors intention, indicating about 82% of the total effects on behavior intention. Likewise, there was a significant indirect effect of knowledge (SPC = 0.34, *p* < 0.001) through enhancing PBC with 18% of the total effects on student’s intention (Fig. 1, Table 5). Among all constructs of planned behavior theory, PBC constructs were the strongest determinant (*p* < 0.001) for behavior intention compared other constructs, indicating about 44% of the total effects on behavior intention (Table 5).

**Table 5:** Direct and indirect effects of theory of planned behavior constructs and knowledge

Determinants or Predictors	Causal Effect		
	Direct	Indirect	Total effects
Knowledge→Intention*	–	0.340	0.340
Attitude →Intention*	0.180	–	0.180
Subjective norms→Intention*	0.147	–	0.147
Perceive behavior control→Intention**	0.830	–	0.830
knowledge→Perceive behavior control**	0.410	–	0.410
Through total causal effect	1.567	0.340	1.907
Percantage of indirect and indirects effects	1.567/1.907 = 82%	0.340/1.907 = 18%	

*p < 0.05, **p < 0.001

## Discussion

This study examined the effects of psychosocial characteristics and individual’s knowledge on adolescent’s intentions to use iron and vitamin D supplements in Iran. Our results showed that socio-demographic variables had no significant effect on behavioral intention. Similar findings were reported previously which found that demographic characteristics exerted little effect on the intention behaviors [37, 40, 45].

Our finding suggests that an individual’s knowledge toward dietary vitamin D and Iron was moderate (4.58 from 9) (Table 1). Most adolescent (60%) in present study were aware of the potential health outcomes (such as calcium absorption, intelligence quotient, and delayed physical growth) associated with Iron and vitamin D deficiency. Likewise, our results showed that most of adolescents (> 50%) were not aware of some preventive effects of vitamin D and Iron supplementation (e, g., anemia and the immunity of the body) common in old age, and the focused on the here and now. This finding were in line with several studies on young adults and adolescents, who have been considered as “young invincible” because of their disregard for future health consequences [46, 47].

Furthermore, testing SEM helped to explain that total effects of knowledge resulting from significant indirect pathways. Our findings showed that indirect effects of individual’s knowledge were negatively mediated by perceived behavior control. The majority of previous studies reported the direct routes of individual’s knowledge on intention behaviors, failed to observe indirect effects [37, 48, 49]. This present work lends some support to the results of an American women and Canadian young adults studies performed by Heather Petraszko (2013) and Goodman (2015), who observed the effects of individual’s knowledge on behavioral intention could be mediated through constructs of perceived behavior control [37, 40].

The results from testing SEM showed good fit between the constructs of TPB and data, indicate all the significant paths were in the expected direction as it was reported by Heather Heather Petraszko (2013) and Goodman (2015) on the frequency of nutrition multivitamin/mineral use among American and Canadian young adults [37, 40]. Interestingly, our finding showed that the constructs of TPB are able to explain 74% of total variance of individual’s intention to use iron and vitamin D supplements. This is in agreement with several studies utilizing the application of TPB to predict nutrition and dietary supplements [48, 50]. Hagger et al. (2010) indicated the predictive relationships between key construct of TPB (i.e., attitude, subjective norms, PBC, intentions) and young adult’s intention to use multivitamin/mineral as well as the frequency of supplementation [51]. Ajzen have suggested that TPB as an appropriate choice to predict and explain people’s intentions and behavior to use fruit, vegetable and nutrition intake [50, 52].

Among all the constructs in this study, PBC emerge as the strongest direct path of intention, that mediates the effects of knowledge on individual’s intention to use dietary supplements among adolescents. Our finding suggests that adolescent who have a higher perception and cognition, show a greater intention to engage in iron and vitamin D consumption on a regular basis despite various conflicting situations. This is consistent with TBP assumptions and other similar studies that have indicated perceived difficulty or ease of performing the behavior significantly influence the individual’s intention to perform the behavior [50, 53, 54].

In present study, attitude positively affected individual’s intention, which was consistent with the TPB. Significantly, around 60% of participants either disagreed or strongly disagreed with the specific attitude statements include “getting enough iron and vitamin D through food and I do not need to eat supplements”. Likewise, close to half of participants believed that these supplements do not cause serious health problems in their body. Thus, most of participants possessed a positive sense and belief concerning the benefits of consumption of dietary supplements. However, some subjects (32%) believed that the use of supplements is harmful for their body and should be provided through food intake. A number of studies have found evidence that a high positive sense and attitude toward healthy behaviors is more likely to promote an individual’s intention regarding personal goals, such as supplement use [37, 51, 53].

We found that subjective norms positively affected intentions; accounting for 13–15% of the explained variance. Our finding indicates that friends, fathers and teachers had a great influence, by acting as role models and giving social support. This result was consistent with other studies that reported social influences, such as parents; friends and family doctors could potentially lead to long-term changes in adolescent’s intentions and behavior [50, 52, 53].

Regarding the main strength in this study is that it is one of the first to generate new finding to identify the potential determinant of supplement consumption among adolescent population, using TPB constructs. Examining the application of psychological theories in different population is important to understand the potential predictors in health promotion behaviors. Furthermore, we explored the factor constructs to find out how they influence intention behavior. This is essential approach for each successful investigation since it provides useful information about the potential constructs that influence individual’s intentions to engage the healthy behavior at specific time,

This study is not without limitations: however, these shortcomings explained here can be applied as guide for other studies in future. Our participants were selected from female adolescent population (aged 12–17) who were living in Iran. Our findings may not be generalizable to younger or older population. Secondly, this result generated from strategies in present study to regular intake Iron and vitamin D information may not be applicable for other dietary supplement, with long-term health implications such as omega-3 and calcium. Therefore, future testing of the TPB with longitudinal data among other populations and dietary supplement would be useful to examine whether results are comparable.

Thirdly, although the use of self-reported questionnaire and a cross-sectional data is common in TPB assessment [53], it may have led to conceptual problems in the causal inferences of the TPB and let to over representation the associations among constructs of TPB. Thus, it must be noted that the predictors likely are less strongly associated with consumption of supplements among adolescent than what was observed in present study.

We acknowledge that this study would have been more effective if the education intervention had been conducted and its effect were measured in changing the adolescents’ intention and behavior. Thus, further study should focus on longitudinal data to assess effects of TPB-based interventions on changing young adolescents’ intention and behavior.

## Conclusions

This study provides support for the effectiveness of TPB and its potential constructs in testing for the determinants of iron and vitamin D supplement intake among adolescent in Iran. We found that knowledge, subjective norms, attitude, and PBC could be potential determinates to explain and predict female adolescent’s intentions regarding vitamin D and iron consumption. PBC was the strongest construct of TPB at predicting people’s intentions to use iron and vitamin D.

### Practice implications

This study suggested that an individual’s knowledge, attitude, subjective norms and PBC may influence the level of intention behavioral to intake dietary supplements. Identification of potential determinants that influence effective behavior change could be useful for health promoters and public health policy educators to better perceived adolescent’s needs and intention to intake dietary supplements.

## Abbreviations

SEM: Structural equation modeling
TPB: Theory of planned behavior
